# Influence of kinematic alignment on femorotibial kinematics in medial stabilized TKA design compared to mechanical alignment

**DOI:** 10.1007/s00402-022-04661-5

**Published:** 2022-10-25

**Authors:** L. Bauer, M. Woiczinski, C. Thorwächter, P. E. Müller, B. M. Holzapfel, T. R. Niethammer, J.-M. Simon

**Affiliations:** Department of Orthopaedics and Trauma Surgery, Musculoskeletal University Center Munich (MUM), University Hospital, LMU Munich, Marchioninistr. 15, 81377 Munich, Germany

**Keywords:** Total knee arthroplasty, Kinematic alignment, Mechanical alignment, Femorotibial kinematics

## Abstract

**Introduction:**

Worldwide more and more primary knee replacements are being performed. Kinematic alignment (KA) as one of many methods of surgical alignment has been shown to have a significant impact on kinematics and function. The aim of the present study was to compare KA and mechanical alignment (MA) with regard to femorotibial kinematics.

**Materials and methods:**

Eight fresh frozen human specimens were tested on a knee rig during active knee flexion from 30 to 130°. Within the same specimen a medial stabilized (MS) implant design was used first with KA and then with MA.

**Results:**

The femorotibial kinematics showed more internal rotation of the tibia in KA compared to MA. At the same time, there was a larger medial rotation point in KA. Both alignment methods showed femoral rollback over the knee bend.

**Conclusion:**

Relating to an increased internal rotation and a more precise medial pivot point, it can be concluded that KA combined with a MS implant design may partially support the reproduction of physiological knee joint mechanics.

**Supplementary Information:**

The online version contains supplementary material available at 10.1007/s00402-022-04661-5.

## Introduction

The knee joint is the second most common joint to be replaced, with 124,677 primary implantations being recorded as of 2020 in Germany alone [1]. However, it is well known that approximately 20% of patients with total knee arthroplasty (TKA) remain dissatisfied after the procedure [2–4]. To enhance the procedural outcome, research is being conducted in various areas of TKA. In addition to the different implant designs, such as cruciate retaining (CR), posterior stabilized (PS) or medial stabilized (MS), there are also differences in surgical techniques and alignment strategies. To be more specific, in mechanical alignment (MA), the proximal tibial cut is made perpendicular to the mechanical tibial axis. This means that a straight leg will be the outcome, regardless of the prearthrotic deformity [5]. Kinematic alignment (KA), on the other hand, involves a three-dimensional approach and attempts to restore the constitutional alignment of the patient. Here, the implant is positioned based on the natural knee joint axes, taking the individual anatomy of the patient into consideration [5, 6]. Both the designs and alignment techniques aim to restore the best possible physiological knee joint movement during flexion and extension, despite their differences. Overall knee joint movement has been described by Pinskerova et al. (2020) as an orchestra of different movements during active flexion including internal tibia rotation, (mainly) lateral femoral rollback, and a hint of varus rotation/tibial adduction [7].

KA has been used in clinical practice for more than 10 years. This accounts for the lack of sufficient empirical data so far, especially in terms of implant survival. The assumption that KA would lead to faster implant failure has been proven wrong or has not yet been confirmed [6]. Moreover, in 2012 one of the first randomized controlled trials comparing KA to MA demonstrated a generally higher patient satisfaction with KA, as shown through the significantly better Knee Society Score results [8]. These results have been confirmed by a later study by Courtney et al. in 2017 [9]. Going further, significantly greater knee flexion, as well as a higher likelihood for pain relief, was also shown in patients who underwent KA [8]. Rivière’s group published a systematic review in 2017, comparing the safety and efficacy of KA and MA. The study revealed that osteoarthritic patients showed faster recovery and higher functional outcomes with KA than those who underwent MA [10]. The retrospective study from Abhari et al. compared the Patient-Reported Outcome Measures (PROMS) from 115 TKA patients, who underwent restricted KA, to a matched group with 115 TKA patients who underwent MA within a one-year follow-up. After implantation, 93% of the KA TKA patients were either very satisfied or (just) satisfied. On the other hand, only 81% of the patients with MA TKA were shown to be satisfied. At one-year follow-up, commonly used PROMS (KSS Knee/Function, WOMAC, and KOOS JR) showed that kinematically aligned patients were significantly more satisfied with their TKA than mechanically aligned patients [11]. Chen et al. compared the biomechanics and long-term wear of KA and MA during walking by using a simulation through a multibody musculoskeletal model [12]. This model estimated an 8.2% increase in the maximum medial tibiofemoral contact force, a maximum posterior translation of 4.7 mm, and a 5.5% decrease in wear volume over a 10-million-cycle simulation for KA group. However, the authors noted that no relevant differences were found between KA and MA in terms of contact mechanics, range of motion, and long-term wear.

However, there remains a lack of data to follow up on the initial positive results of KA. Biomechanical in vitro studies are strongly needed to provide objective preclinical data that could prove whether one alignment method is superior to the other, or if certain anatomical conditions should qualify for exclusion criteria. Moreover, most of the currently designed knee implants were originally not developed for their use in combination with KA.

We hypothesize that KA TKA is closer to the native situation regarding kinematics than MA TKA.

## Methods

### Human specimen and implantation

Eight fresh frozen human specimens were used: four females (two right, two left knees) and four males (two right, two left knees). Patients from whom the specimens had been obtained had a mean age of 79.6 (± 5.9) years. The patients were otherwise healthy and had a clinically determined straight axis of the leg. The specimens were thawed at room temperature 24 h before the start of the experiment. The skin and unnecessary tissue were removed. Only the tendons of the M. quadriceps femoris, M. biceps femoris, and M. semitendinosus were retained. They were fastened into finger traps using medical suture material. Based on the total leg length, the mechanical axis of the leg was drawn over the femoral head and the ankle joint. Finally, the tibia and femur were shortened to 22 cm and 20 cm length (as measured from the epicondylar axis), respectively. The fibula head was fixed to the tibia with a cortical screw to ensure stability during the test-setup. The ends of the tibia and femur were embedded into metal cups using epoxy resin to clamp the knee joint into the knee rig [13–18]. To secure a malalignment of the femoral bone within the metal cups, the internal/external rotation of the posterior condyles of the femur were aligned parallel to the hip flexion axis of the experimental setup in the transversal plane.

For knee joint replacement, the GMK Sphere (Medacta International, Castel San Pietro, Switzerland) total knee system was applied, using a MS polyethylene insert. All experiments were started with KA followed from MA alignment. To be able to convert after KA to MA, primarily, the femoral distal cut reference was set by inserting two pins to fixate the distal cutting block with a 6° valgus correction measured from the anatomical axis to achieve an appropriate biomechanical axis within MA while using the Medacta GMK Sphere implant in the first place. From now on, the kinematic alignment was performed, using a distal cut reference labeled “worn” and contact to both the femoral condyles for KA, respecting the native joint axis. As a result, due to the marking of the distal femoral cut for both KA and MA, a simple and steady conversion was made possible. First, the GMK Sphere with an MS insert was implanted using the KA technique. Corresponding to the thickness of the distal condyles of the femoral implant, a distal femoral cut of 9 mm was performed. The femoral rotation was determined by using the posterior condyle tangent line without any correction to obtain the preexisting flexion gap. The resected bone material from the distal femur cut (medial and lateral condyle) was preserved for the switch to the MA technique later. The tibial cut was performed based on the specimen-specific joint line and slope. Subsequently, the same TKA system was implanted using the MA technique. Therefore, the distal femoral resection bone material (medial and lateral condyle) was pinned back to the femoral condyles using a Kirschner’s wire. Afterwards, the distal cutting block was fixed using the previously set two pin holes respecting the mechanical leg axis with a 6° valgus correction, as described above. To achieve a correct distal cutting amount the cutting block was moved proximally 2–4 mm using the repositioning holes. This was necessary to achieve a sufficient resection amount on both medial and lateral condyles due to correction of the joint line in the context of MA. To avoid a potential bias by modifying the femoral rotation in line with MA, the femoral rotation remained the same as within KA. Hence, all the other femoral cuts (anterior, posterior, and chamfer) remained the same. The tibial cut was applied using the intramedullary alignment system with a standard 3° tibial slope. To achieve a sufficient tibial resection amount, the cutting block was distalized by 2–4 mm. Similarly, this allowed for a stable flexion and extension gap, due to a narrowed distal femoral cut within MA. Consequently, we accepted a distalization of the joint line by 2–4 mm within MA. For all implantations (KA/MA), the same insert size (10 mm thickness) was used, allowing for sufficient ligament stability. However, for MA no soft tissue release was performed. The range of the implant sizes was 3–6 for the femoral and 4–5 for the tibial components. All the implantations were performed by two surgeons (JMS and TRN) familiar with the implant system.

Nano-computer tomography was performed for each specimen before and after implantation to verify implant positioning and to measure the amount of posterior tibial slope.

### Experimental setup

**Fig. 1:**
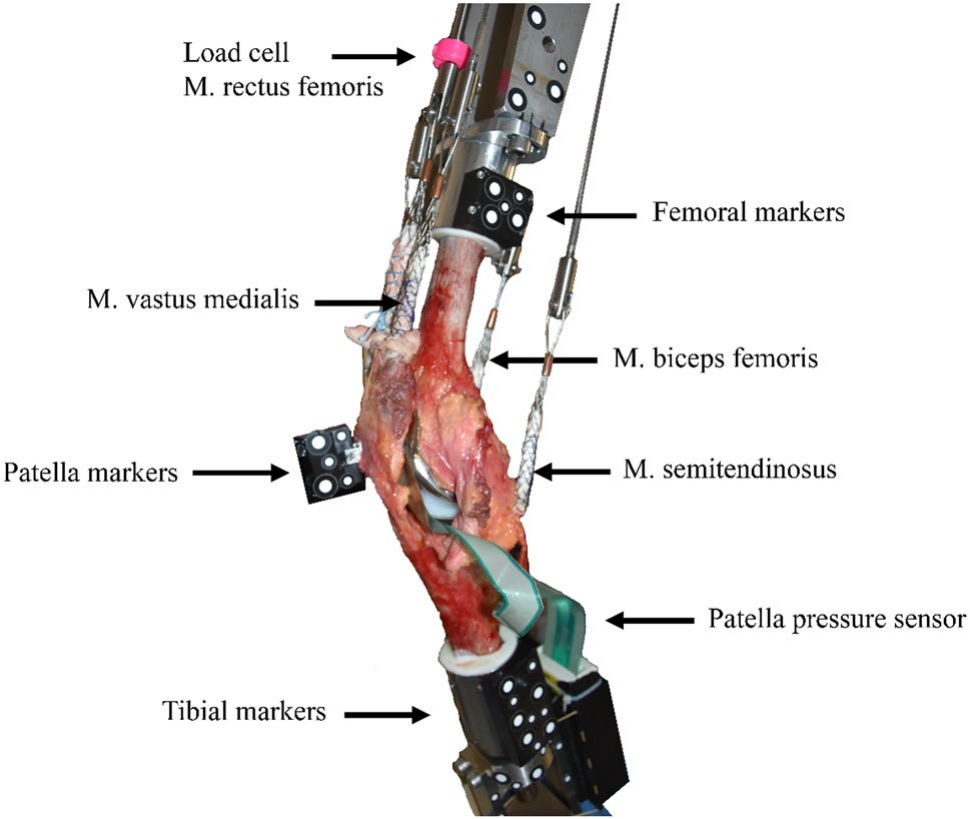
Experimental setup for human specimen with the total knee prosthesis and markers for optoelectrical measuring system

The established Munich knee rig, which has six degrees of freedom, performed an active movement of 30–130° of flexion in the knee joint at a speed of 3°/s [13–18]. The experimental setup does not allow movement at 0° flexion due to the uncontrolled hamstring muscles which normally induce the flexion movement. A constant ground reaction (GRF) force of 50 N was applied during the entire movement. The constant GRF was controlled by the rectus femoris muscle. The activity of the vastus medialis, vastus lateralis, semitendinosus, and biceps femoris muscles was simulated by means of 2 kg weights attached to the tendons. The quadriceps force was measured via a sensor on the tendon (8417-6002 Burster, Gernsbach, Germany). The test was controlled via LabView in real time (Version 8.6, National Instruments, Austin, Texas, USA). Optical markers were attached to the femoral and tibial heads, as well as to the patella (Fig. 1). An optoelectrical measuring system (ARAMIS 3D Camera 2.3 M, GOM GmbH, Braunschweig, Germany) was used to record the movement of the specimen.

Since the Munich knee rig can apply a constant ground reaction force of 50 N, which might lead to a reduced compression force femorotibial compared to in vivo loading, an additional test was carried out to investigate which shear forces would result from different axial forces for an AP motion, especially in a modern medial highly congruent design. Therefore, the implant (GMK Sphere, Medacta International, Castel San Pietro, Switzerland) was mounted in a testing machine (ElectroPuls™ E10000, Instron, Norwood, USA) and loaded with different constant axial compression forces (25 N–300 N). The tibial component was displaced 2 mm anteriorly and the shear forces were measured.

### Data analysis

Data were analyzed with MATLAB (MathWorks Inc., Natick, MA, USA). The data from the optoelectrical measuring system were synchronized and interpolated with the flexion angle and recorded by the knee rig program.

**Fig. 2:**
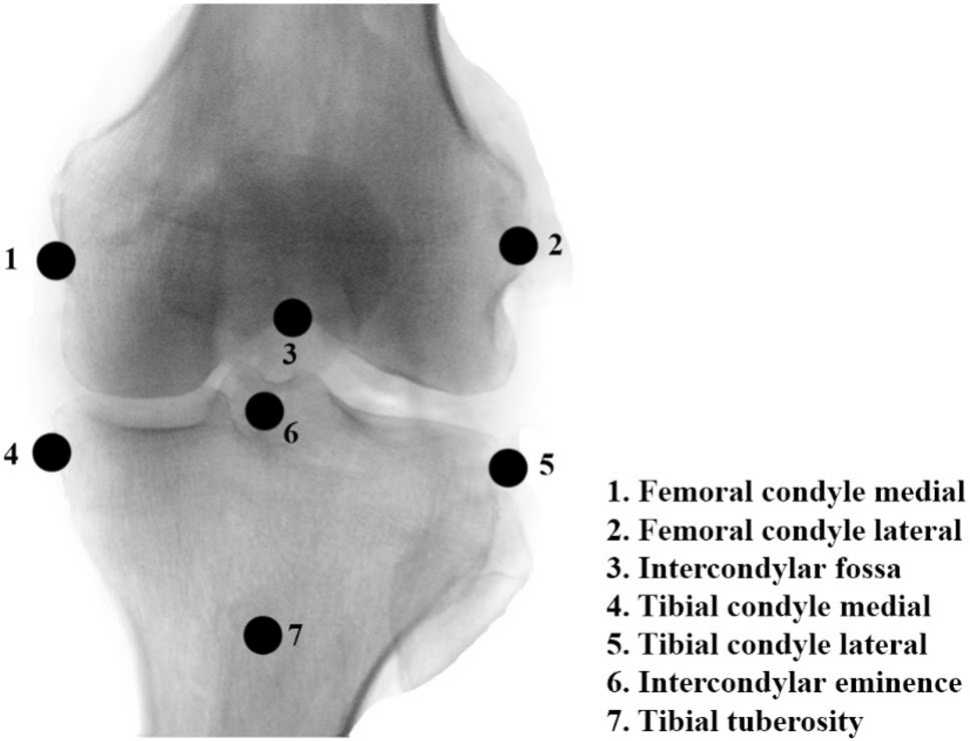
Landmarks of the femur and tibia used for the calculation of femorotibial kinematics

The kinematics of the femorotibial joint (tibia rotation and anterior–posterior tibia movement) was calculated using the motion data provided by an optoelectrical measuring system. A well-established method was used that has been previously described in detail [19, 20]. Various landmarks of the knee joint were used for this calculation (see Fig. 2). The central AP motion is a translation of the intercondylar fossa (Point 3). Points 1 and 2 (Fig. 2) were used as the flexion facet centers of the posterior condyles and projected onto the insert/tibial plateau [21]. These points were connected graphically and projected to the tibia plateau to represent the rotation of the knee. This was done through flexion increments of 5°/10° from 30° to 120°. Referenced to the positions at 30° knee flexion, the straight lines were averaged over all preparations and the standard deviation was given in both the *x*- and *y*-directions.

The results shown here are the mean across eight knee joints with a 95% confidence interval (CI). For statistical analyses SPSS (IBM SPSS Statistics 27) was used. For this purpose, the values at 30°, 60°, 90°, and 120° flexion for the native situation, the KA, and MA were analyzed by means of one-factor analysis of variance (ANOVA). The results were presented with boxplots. The significance level was set at 0.05.

## Results

The implanted prosthesis had a posterior tibial slope of 2.31 (± 0.86)° after kinematic alignment, which also reflects the native situation. After mechanical alignment, a posterior tibial slope of 2.94 (± 1.4)° was achieved.

**Fig. 3:**
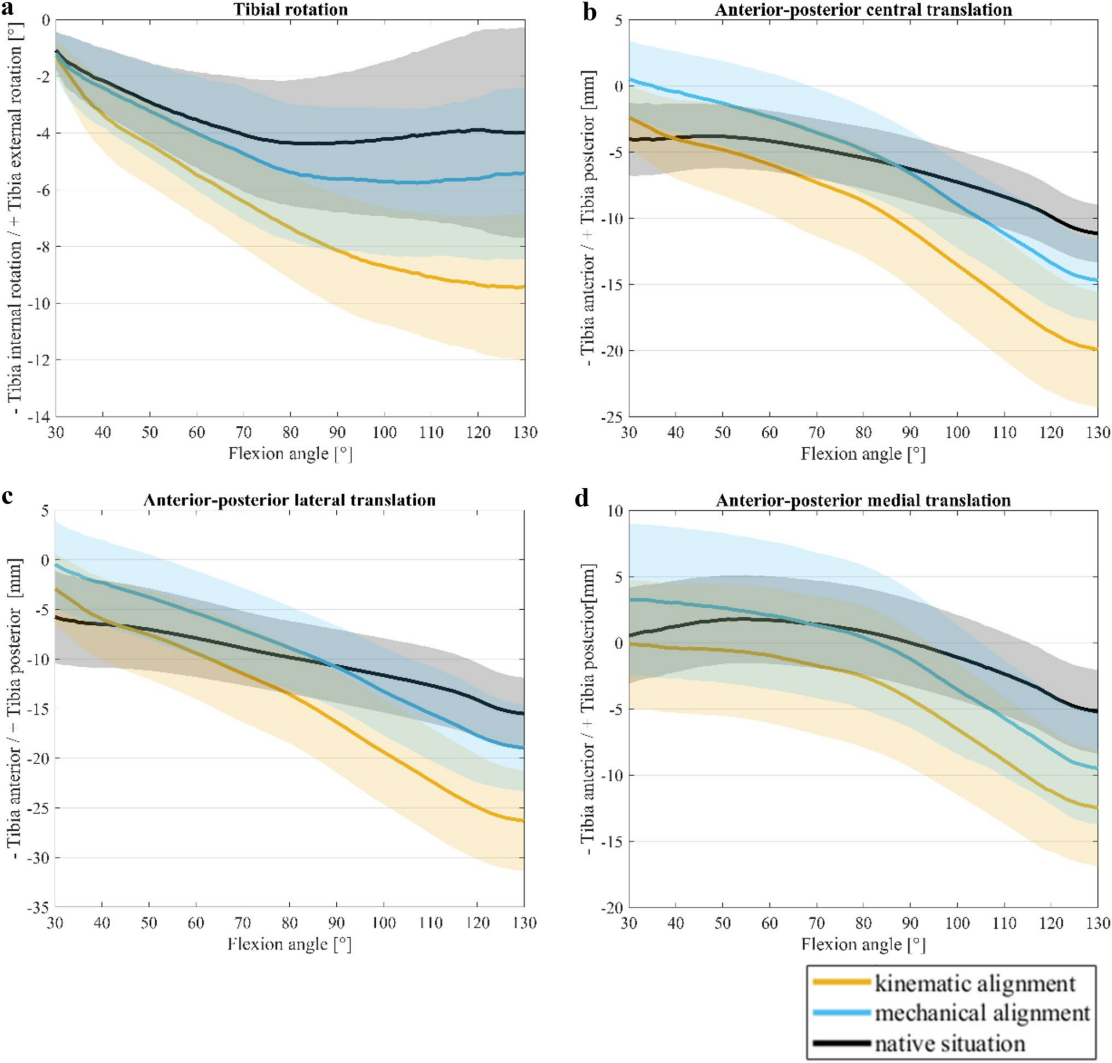
Femorotibial kinematics of the knee joint with mechanical alignment, kinematic alignment and native situation. Mean values (*n* = 8) and 95% confidence interval of femorotibial kinematics of a medial stabilized prosthesis during flexion from 30° to 130°; **a** Tibial rotation, **b** Anterior-posterior central translation, **c** Anterior-posterior lateral translation, **d** Anterior-posterior medial translation, for kinematic alignment (orange), mechanical alignment (blue) and the native situation (black)

Figure 3a shows the rotation of the tibia. Initially (30° flexion), no difference in rotation was observed. With increasing flexion, the tibia of KA rotated increasingly inwards. From approximately 90° of flexion, the slope of the curve for internal rotation decreased slightly but showed further progress. With MA, the tibia rotated less inward. The curve flattened in MA from 80° and the tibia hardly rotated during deep flexion (80–130°). From 90° flexion, significantly less internal tibial rotation was visible with MA than with KA. The least tibial rotation during flexion of the knee joint was seen in the native situation. The internal rotation curve flattens out from 70° flexion and stagnates until deep flexion. Figure 3b shows the AP movement of the tibia (relative to a fixed femur). This movement was represented by the tracked landmark intercondylar fossa (see Fig. 2, landmark point 3). With KA, the tibia was already located more anteriorly at the beginning compared to MA (2.9 mm). Over the flexion range of the knee joint (30–130°), a similar course for KA and MA was observed for the AP central movement. Both implantation techniques led to an anterior movement of the tibia which means a femoral rollback. Following this, a similar relationship between MA and KA was visible during AP movement on the medial side (see Fig. 2, landmark point 1) and lateral side (see Fig. 2, landmark point 2). For lateral AP movement, a steady increase in the anterior translation of the tibia until deep flexion was detected (see Fig. 3c). In contrast, for the AP movement of the medial side (see Fig. 3d), less translation was visible at lower degrees of flexion (30–60°) although more translation of the tibia was visible anteriorly at higher flexion angles (80–130°). In the native situation, less movement in the AP area was detected (see Fig. 3b–d).

**Fig. 4:**
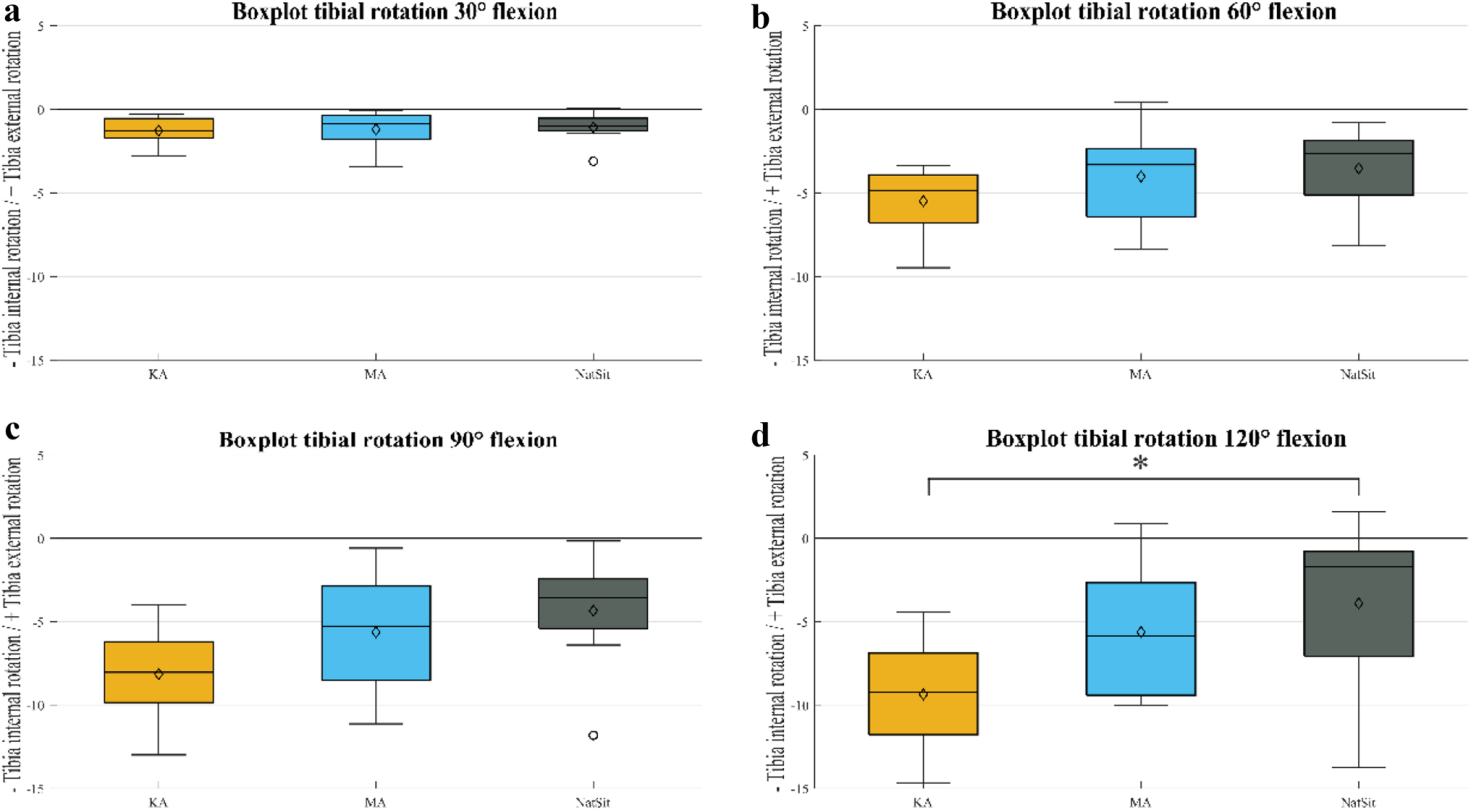
Boxplots of tibial rotation for 30°, 60°, 90° and 120° of knee flexion. Boxplots of tibial rotation showing the median (*n* = 8), first and third quartile, range and outliners for kinematic alignment (KA, orange), mechanical alignment (MA, blue) and the native situation (NatSit, gray); for **a** 30° of flexion, **b** 60° of flexion, **c** 90° of flexion and **d** 120° of flexion. Mean values are marked with ◇; Significant differences (*p* < 0.05) are marked with asterisks

Figure 4a–d shows boxplots for the tibial rotation at KA, MA and the native situation (NatSit). These include the median, the first and third quartiles, as well as the range. The outliers are shown with dots. A square bracket with an asterisk indicates a significant difference in the values (*p* < 0.05). It can be seen that the median tibial rotation decreases with increasing flexion (30–120°), reflecting increased tibial internal rotation. The length of the boxes for KA and MA increased, representing a wider divergence of the data. At 120° flexion, the median was close to the third quartile, showing skewness in the data distribution. At this point of flexion, the tibial rotation for KA and the NatSit differed significantly from each other (*p* = 0.05). Further boxplots, corresponding numbers and p-values can be seen in the appendix (Appendix Figs. 6–8 and Tables 2–5).

**Fig. 5:**
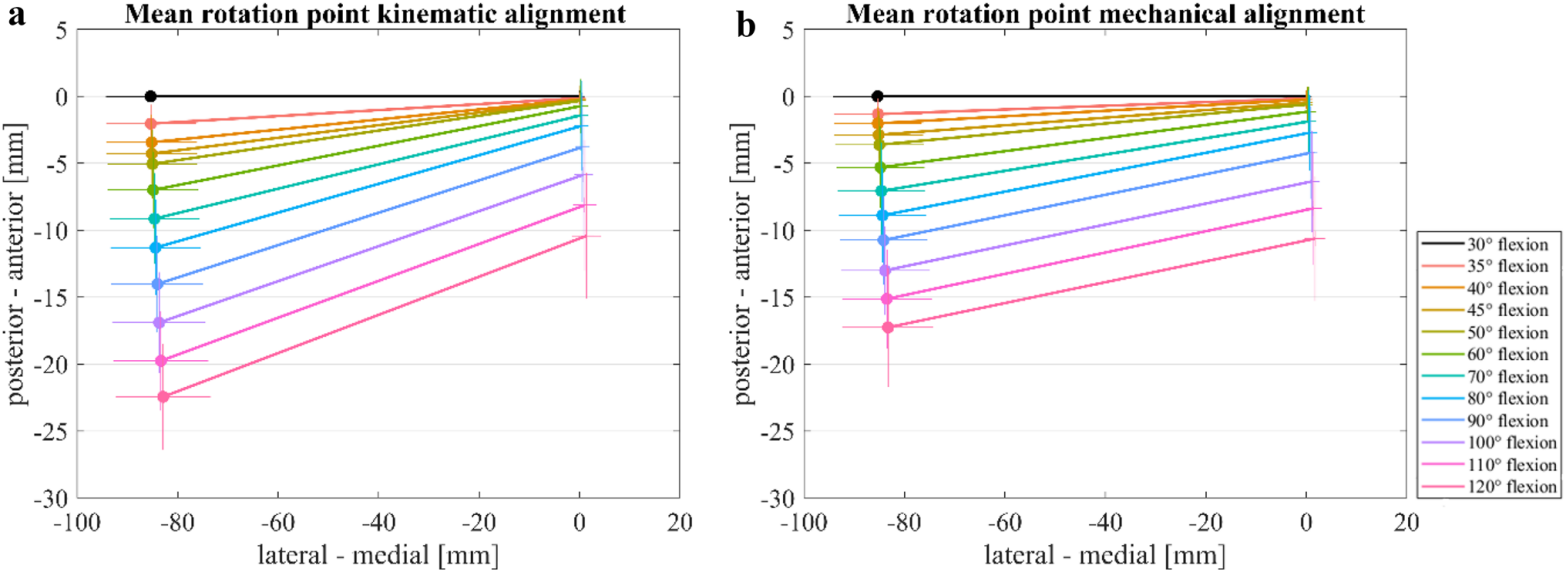
Medial rotation points of the knee flexion with a total knee prosthesis. Mean value (*n* = 8) of the medial rotation point and horizontal/vertical standard deviation of **a** kinematic alignment 30–120° flexion, **b** mechanical alignment 30–120° flexion

Figure 5a–b represents the projection of the femoral condyles at the surface of the insert to interpret the pivot point between the femur and tibia. In Fig. 5a, the lateral (o) and medial (+) condyles were imaged at 30–50° flexion in 5° steps and 50–120° of flexion in 10° steps for KA and a rotation point around the medial condyle (medial pivot) became visible. Thereby, a continuing rotation point up to approximately 70–80° of flexion was shown for KA. From 80° of flexion, a translation of the connecting line from the medial and lateral point to posterior was observed. In Fig. 5b, flexion is shown in the same flexion steps as 5a from 30 to 120° of flexion for MA. A medial rotation point was also shown for MA. However, this was less pronounced since less movement was visible on the lateral side. Moreover, at higher degrees of flexion, the medial rotation point was less clearly visible and an increased posterior translation of both points (medial and lateral) was noticeable.

For the additional information on the required shear force for an AP movement of 2 mm at different axial forces for the tibiofemoral compressive force of 250 N, only 25.21 (± 0.06) N shear force was measured (Table 1).

**Table 1:** Required shear force for AP movement of 2 mm for different axial forces

Applied axial force [*N*]	Required shear force [*N*]
25	3.57 (± 0.14)
50	6.77 (± 0)
100	13.20 (± 0.24)
150	18.73 (± 0.26)
200	22.09 (± 0.11)
250	25.21 (± 0.06)
300	28.27 (± 0.15)

## Discussion

The main findings of the present study were a greater extent of tibial internal rotation and femoral rollback (AP translation of the tibia) during flexion for KA TKA compared to MA TKA. Equally, for KA, the, due to the inlay design expected, medial pivot point is more precise accompanied by a greater lateral AP movement.

A more precise medial pivot point is associated with a higher level of tibial internal rotation and enables a sufficient lateral femoral rollback as seen within KA TKA. This is consistent with the physiological knee kinematics described by Pinskerova et al. [7]. However, these findings cannot be transferred to our native kinematics that was measured with the knee rig. Concerning this matter, our results show a noticeably reduced femoral rollback and tibia internal rotation, especially between 80 and 130° of flexion in the native situation. This corresponds to the findings of Dupraz et al. [22], who used the same knee rig, and of Varadarajan et al. [23] who used the Oxford knee rig which is similar to the rig used in this study. The reduced tibia rotation is consistent with the findings of Varadarajan et al. regarding the ability of in vitro systems to capture the characteristic differences of the flexion–extension kinematics of the native and replaced knee joint. They suggested that the differences between in vivo and in vitro tibia rotation might arise from the difference between the simulated and the in vivo muscle forces but, in vitro systems such as the weight-bearing knee rig can simulate the key kinematic feature of the native knee [23]. The findings of the present study are consistent with the findings of Maderbacher et al. who evaluated the tibiofemoral kinematics of healthy knees, KA and MA TKA within a flexion angle up to 90°. They demonstrated that KA TKA allows for more physiological tibial internal rotation and femoral rollback than MA TKA [24]. This was underlined by an even greater tibial internal rotation and femoral rollback within the healthy native knee. As a limiting factor, they only performed a mere passive motion of the knee joint while using a continuous passive motion device.

Regarding the starting point of the flexion movement, the KA TKA showed a closer association to the native knee situation. Within the MA TKA, we were able to see a more posterior position of the tibia when starting the flexion movement. This might be attributed to the fact that for MA TKA, the tibial slope is set to a level of 3°, whereas with KA TKA, the native patient-specific slope is maintained. In this regard, our results showed that maintaining the native tibial slope within KA TKA might contribute to more internal tibial rotation knee kinematics after arthroplasty. After MA TKA, we performed CT scans of the specimen in order to reevaluate the correct position of the implant. On average, the intended 3° posterior tibial slope was well met with an actual slope of 2.94 (± 1.4) °. This assumption is confirmed by a study of Fujito et al. who stated that a higher amount of posterior tibial slope (≥ 8°), compared to a tibial slope ≤ 7° after TKA, generates a greater range of motion and a higher maximum flexion angle of the knee [25]. However, within their findings, the posterior tibial slope did not affect the external rotation angles and the AP position.

In this regard, we used an MS insert for TKA which has an asymmetric shape with a limited AP translation on the medial side and an unrestricted translation on the lateral side, comparable to native knee joint kinematics [26]. Our results show that the effect of this medial pivot design on functional parameters is even more pronounced when implanted using KA: a more precise medial pivot point with a greater extent of lateral AP translation. This is supported by two clinical studies of Risitano et al. and Sabatini et al. who examined the clinical outcomes (Oxford Knee Score, Knee Society Score) of patients who underwent KA TKA, with the use of a medial pivot design. Both their analyses showed good to excellent postoperative clinical results within a one-year follow-up, with a patient satisfaction of up to 95% and an average maximum active flexion of 124° [27, 28]. Furthermore, a study by Alesi et al. also found out that a better medial pivot point in vivo was associated with higher patient reported outcomes. Also in these studies it was shown that there was a slight movement of the femoral condyle medially [29]. In our study this clear pivot point is especially visible in the KA knees and would therefore suggest to prefer KA in medial stabilized knee designs. It was shown that in an experimental test setup, such as the Knee Rig in this study, the greatest difference in kinematics resulted between a passive, non-weight-bearing and an active, weight-bearing setup. The magnitude of the applied GRF is not reflected in different kinematic trajectories over the flexion cycle, but only in the magnitude of the absolute values [30]. However a reduced GRF compared to in vivo loading might have an influence on femorotibial compression, and therefore might especially affect the femorotibial kinematics in a medial stabilized (ball and socket) insert design. To verify this mechanism, an additional anterior/posterior test, as described above, was performed. This test showed that at a tibiofemoral compressive force of 250 N, only 25.21 (± 0.06) N shear force was required to displace the femoral component 2 mm from its position. In the Bergmann data, 250% of the body weight represented the resulting compression force in the joint for a deep knee bend [26]. Being transferred to our simulated patient situation (knee rig) with 50 N per side (corresponds to a total weight of 100 N), 250 N had to be applied for this additional test. Bergmann et al. also described a shear force in the anterior or posterior direction of ± 10% of body weight for a deep knee bend. Overall, this could be an explanation for the slightly medial AP movement and should be considered, especially when testing highly congruent inserts in deep knee flexion. Whereas even within in vivo studies which could show a clear medial pivot rotation point, AP movement was visible on the medial side [29]. Altogether, this shows that a constant ground reaction force of 50 N is sufficient to simulate deep knee flexion and the resulting tibiofemoral contact force that allows a slight translation out of the ball and socket.

However, it is worth noting that the present study has some limitations. Firstly, this was an in vitro study with a small number of samples. This means that the results cannot be directly transferred to the in vivo situation. However, it is inevitable to take a closer look at the biomechanical differences in a laboratory experiment. In patient studies, the exact measurement of kinematics is not possible. A case number of 7–10 specimens is widely used in this research area. Therefore, we included eight specimens in our study. The Munich knee rig used, with its active knee flexion controlled by the rectus femoris muscle, is a very good simulation option for in vitro purposes. However, the experiments were carried out with a constant ground reaction force of 50 N. The author group suspects that this GRF in deep flexion have to be considered carefully, especially for a MS insert. An experimental setup with too low compression force may increase posterior translation of the medial condyle when looking at the pivot point. Due to the lack of force from below, the femur could push out slightly from the medial stabilizing point. However, additional tests within our study and comparison to in vivo data of Bergmann et al. [26] showed a reliable setup within the Munich knee rig. Another limitation was the nature of the specimens. Healthy knee joints were used to guarantee comparability. Yet, the severity of the pathology is particularly important in KA, since the degree of osteoarthritis influences the implantation process. Therefore, this could mean that the differences between KA and MA might even be greater in clinical applications. In addition, the legs had to be shortened to perform the experiments, so the different alignments affecting different angles in the knee joint could not be verified postoperatively. By the resection of the distal femoral cut for the transfer to MA, it cannot be completely ruled out that a slight joint laxity was present, which would affect the kinematics. The use of a different insert, for example a cruciate-retaining insert which is less medial stabilizing, may lead to an overall higher medial tibial translation and thus also have a certain influence on femorotibial kinematics.

The KA TKA showed an adequate amount of femoral rollback laterally, a greater internal tibia rotation and a clear medial pivot point compared to the MA TKA. In this regard compared with clinical investigations [7, 29], it can be concluded that KA TKA using a MS insert design may support the reproduction of physiological knee joint kinematics, which leads to a better postoperative patient reported outcome.

## Supplementary Information

Below is the link to the electronic supplementary material.

Supplementary file1 (DOCX 371 KB)
