# Supplementary material for: Influence of kinematic alignment on femorotibial kinematics in medial stabilized TKA design compared to mechanical alignment
Source: Arch Orthop Trauma Surg. 2022 Oct 25;143(7):4339–47. doi: 10.1007/s00402-022-04661-5 (PMC10293425; doi:10.1007/s00402-022-04661-5)
Supplement: Supplementary file 1 — Supplementary file1 (DOCX 371 KB) [file 402_2022_4661_MOESM1_ESM.docx]

**Appendix**

Table 1: Mean values (n = 8) and standard deviation (SD) for tibia rotation of 30°, 60°, 90° and 120° for kinematic alignment (KA), mechanical alignment (MA) and native situation (NatSit). Significant differences are marked with asterisks (p < 0.05).

| **Tibial rotation**  - Tibia internal / + Tibia external [°] | | KA | MA | NatSit |
| --- | --- | --- | --- | --- |
| **30° flexion [Mean (± SD)]** | | **-1.28 (± 0.85)** | **-1.20 (± 1.13)** | **-1.09 (± 0.94)** |
|  | p-Value to KA | - | 0.99 | 0.92 |
|  | p-Value to MA | 0.99 | - | 0.97 |
| **60° flexion [Mean (± SD)]** | | **-5.49 (± 2.16)** | **-4.02 (± 2.91)** | **-3.53 (± 2.43)** |
|  | p-Value to KA | - | 0.48 | 0.29 |
|  | p-Value to MA | 0.48 | - | 0.92 |
| **90° flexion [Mean (± SD)]** | | **-8.15 (± 2.85)** | **-5.63 (± 3.64)** | **-4.35 (± 3.53)** |
|  | p-Value to KA | - | 0.31 | 0.08 |
|  | p-Value to MA | 0.31 | - | 0.72 |
| **120° flexion [Mean (± SD)]** | | **-9.67 (± 3.42)** | **-5.63 (± 4.11)** | **-3.90 (± 5.03)** |
|  | p-Value to KA | - | 0.21 | 0.05* |
|  | p-Value to MA | 0.21 | - | 0.70 |

**
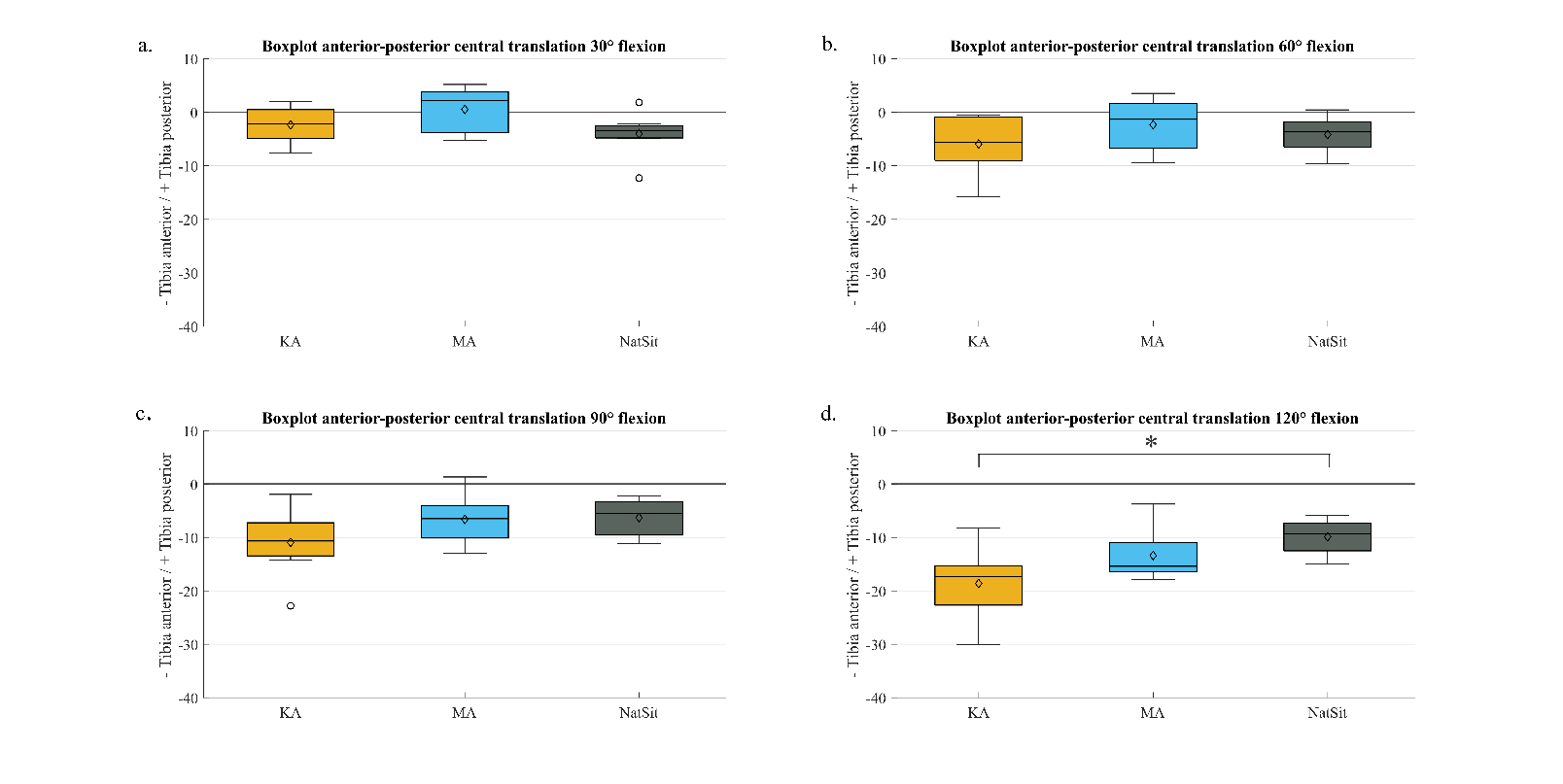
***Figure 6: Boxplot of anterior-posterior central showing the median, 1st and 3rd quartile, range and outliners as points for kinematic alignment (KA, orange), mechanical alignment (MA, blue) and native situation (NatSit, grey); for a. 30° of flexion, b. 60° of flexion, c. 90° of flexion and d. 120° of flexion. Mean values are marked with* ◇; *Significant differences (p<0.05) are marked with asterisks; n = 8.*

Table 2: Mean values and standard deviation (SD) for anterior-posterior (AP) central of 30°, 60°, 90° and 120° for kinematic alignment (KA), mechanical alignment (MA) and native situation (NatSit), significant differences are marked with asterisks (p < 0.05).

| **AP central**  - Tibia anterior / + Tibia posterior [mm] | | KA | MA | NatSit |
| --- | --- | --- | --- | --- |
| **30° flexion [Mean (± SD)]** | | **-2.37 (±3.42)** | **0.51 (± 4.19)** | **-4.03 (±3.95)** |
|  | p-Value to KA | - | 0.32 | 0.67 |
|  | p-Value to MA | 0.316 | - | 0.07 |
| **60° flexion [Mean (± SD)]** | | **-5.94 (±5.43)** | **-2.35 (± 4.75)** | **-4.15 (± 43.30)** |
|  | p-Value to KA | - | 0.28 | 0.72 |
|  | p-Value to MA | 0.28 | - | 0.71 |
| **90° flexion [Mean (± SD)]** | | **-10.92 (± 6.22)** | **-6.61 (± 4.60)** | **-6.29 (± 3.47)** |
|  | p-Value to KA | - | 0.21 | 0.17 |
|  | p-Value to MA | 0.21 | - | 0.99 |
| **120° flexion [Mean (± SD)]** | | **-18.61 (± 6.52)** | **-13.61 (±4.84)** | **-9.88 (± 3.33)** |
|  | p-Value to KA | - | 0.12 | 0.01* |
|  | p-Value to MA | 0.12 | - | 0.37 |

*
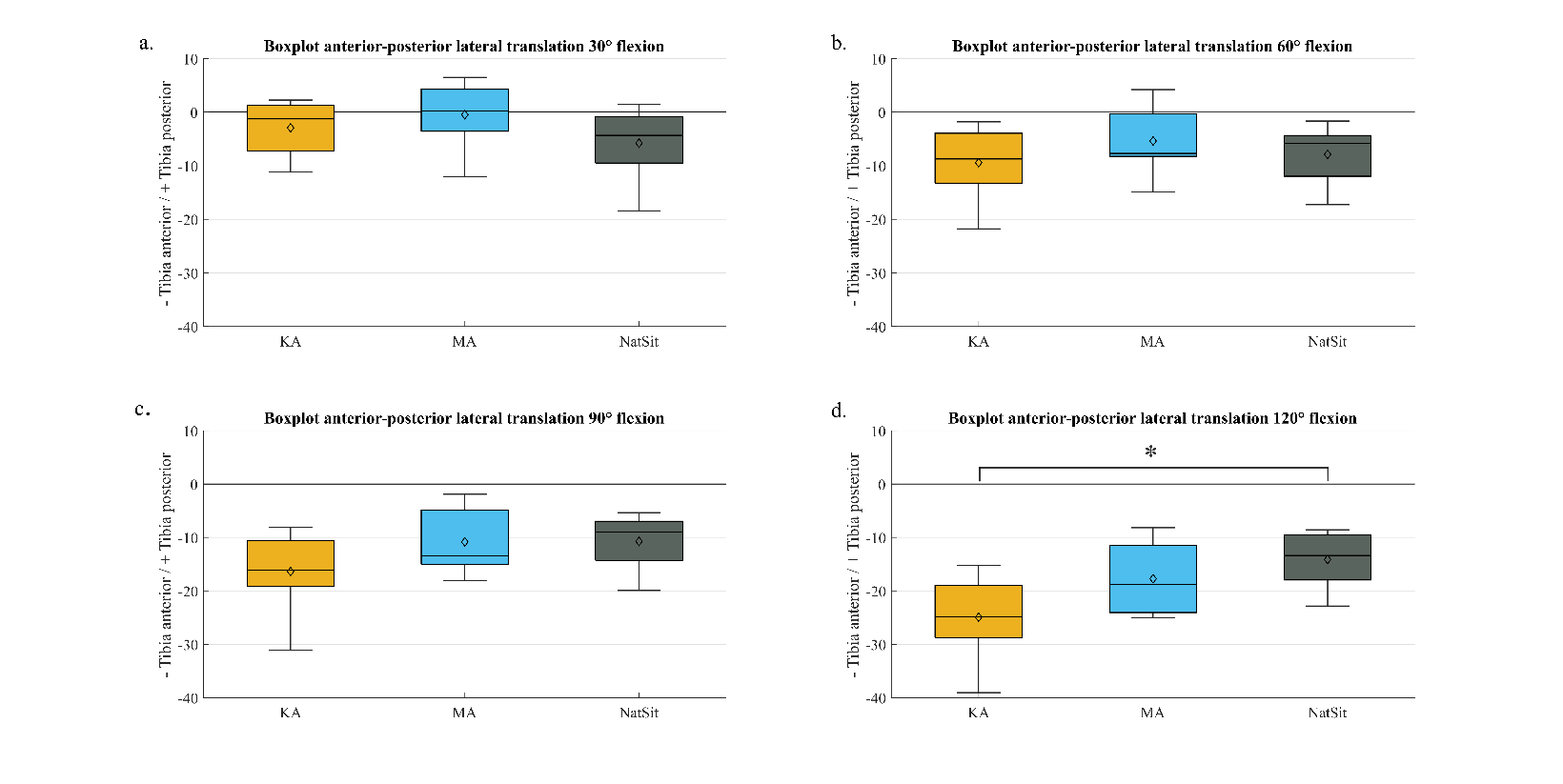
Figure 7: Boxplot of anterior-posterior lateral showing the median, 1st and 3rd quartile, range and outliners as points for kinematic alignment (KA, orange), mechanical alignment (MA, blue) and native situation (NatSit, grey); for a. 30° of flexion, b. 60° of flexion, c. 90° of flexion and d. 120° of flexion. Mean values are marked with* ◇;  *Significant differences (p<0.05) are marked with asterisks; n = 8.*

Table 3: Mean values (n = 8) and standard deviation (SD) for anterior-posterior (AP) lateral of 30°, 60°, 90° and 120° for kinematic alignment (KA), mechanical alignment (MA) and native situation (NatSit). Significant differences are marked with asterisks (p < 0.05).

| **AP lateral**  - Tibia anterior / + Tibia posterior [mm] | | KA | MA | NatSit |
| --- | --- | --- | --- | --- |
| **30° flexion [Mean (± SD)]** | | **-2.92 (± 5.12)** | **-0.48 (± 6.33)** | -5.77 (**± 6.72)** |
|  | p-Value to KA | - | 0.71 | 0.62 |
|  | p-Value to MA | 0.71 | - | 0.22 |
| **60° flexion [Mean (± SD)]** | | **-9.42 (± 6.69)** | **-5.38 (± 6.15)** | **-7.88 (± 5.67)** |
|  | p-Value to KA | - | 0.41 | 0.87 |
|  | p-Value to MA | 0.41 | - | 0.70 |
| **90° flexion [Mean (± SD)]** | | **-16.36 (± 7.33)** | **-10.83 (± 6.02)** | **-10.72 (± 5.34)** |
|  | p-Value to KA | - | 0.21 | 0.20 |
|  | p-Value to MA | 0.21 | - | 0.99 |
| **120° flexion [Mean (± SD)]** | | **-24.93 (± 7.59)** | **-17.73 (± 6.84)** | **-14.12 (± 5.37)** |
|  | p-Value to KA | - | 0.10 | 0.01* |
|  | p-Value to MA | 0.10 | - | 0.54 |

*
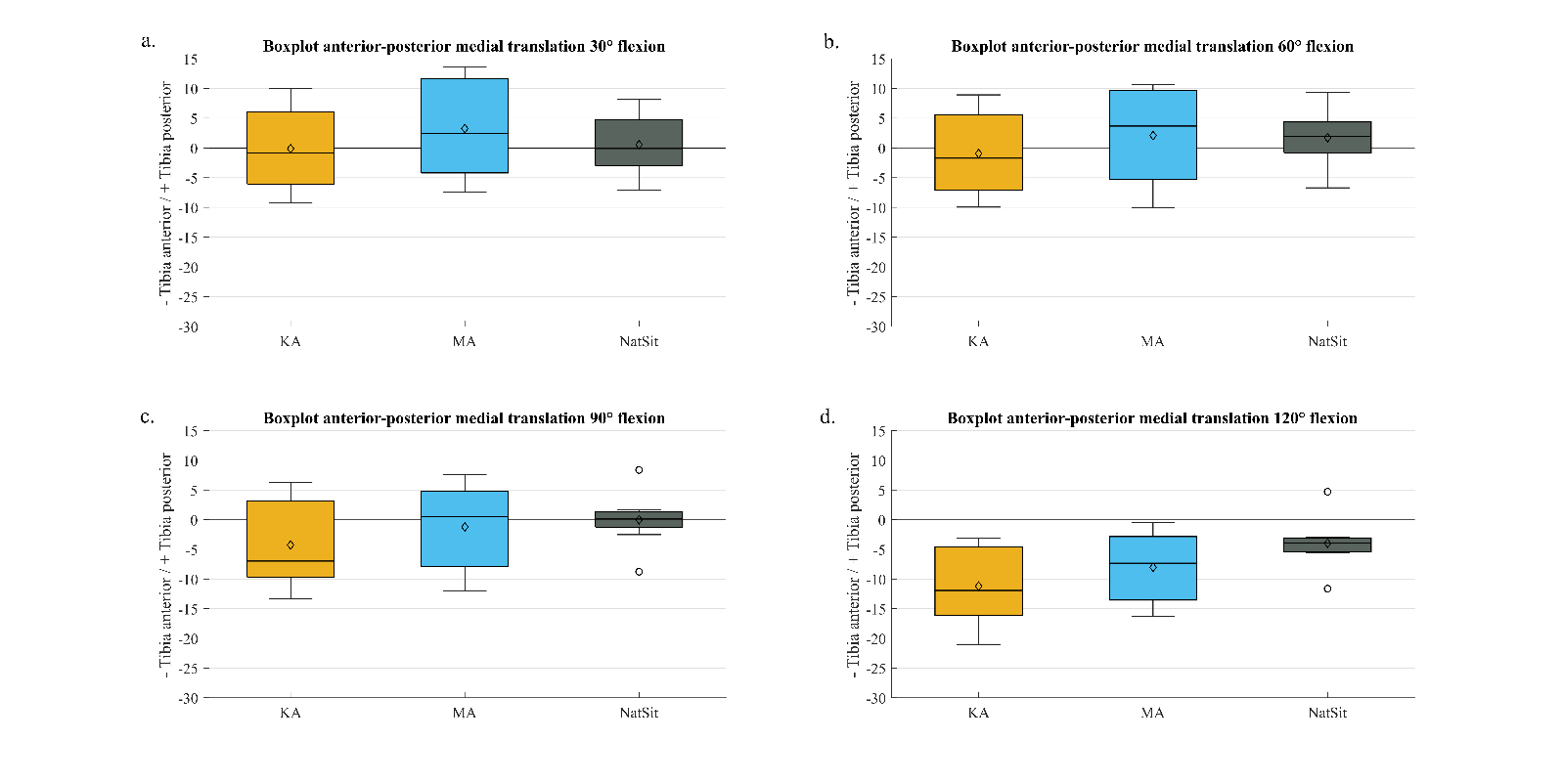
Figure 8: Boxplot of anterior-posterior central showing the median, 1st and 3rd quartile, range and outliners as points for kinematic alignment (KA, orange), mechanical alignment (MA, blue) and native situation (NatSit, grey); for a. 30° of flexion, b. 60° of flexion, c. 90° of flexion and d. 120° of flexion. Mean values are marked with* ◇; *Significant differences (p<0.05) are marked with asterisks; n = 8.*

Table 4: Mean values (n = 8) and standard deviation (SD) for anterior-posterior (AP) medial of 30°, 60°, 90° and 120° for kinematic alignment (KA), mechanical alignment (MA) and native situation (NatSit). Significant differences are marked with asterisks (p < 0.05).

| **AP medial**  - Tibia anterior / + Tibia posterior [mm] | | KA | MA | NatSit |
| --- | --- | --- | --- | --- |
| **30° flexion [Mean (± SD)]** | | **-0.13 (± 7.39)** | **3.25 (± 8.29)** | **0.53 (± 5.22)** |
|  | p-Value to KA | - | 0.60 | 0.98 |
|  | p-Value to MA | 0.60 | - | 0.72 |
| **60° flexion [Mean (± SD)]** | | **-0.95 (± 7.39)** | **2.07 (± 8.07)** | **1.70 (± 4.77)** |
|  | p-Value to KA | - | 0.66 | 0.73 |
|  | p-Value to MA | 0.66 | - | 0.99 |
| **90° flexion [Mean (± SD)]** | | **-4.26 (± 7.43)** | **-1.23 (± 7.50)** | **-0.15 (± 4.73)** |
|  | p-Value to KA | - | 0.64 | 0.43 |
|  | p-Value to MA | 0.64 | - | 0.93 |
| **120° flexion [Mean (± SD)]** | | **-11.20 (± 6.60)** | **-8.02 (± 6.05)** | **-3.98 (± 4.47)** |
|  | p-Value to KA | - | 0.52 | 0.052 |
|  | p-Value to MA | 0.52 | - | 0.36 |
